# Experimental VUV Photoionization of C_70_ and Vibrationally Resolved Spectra of the Excited Electronic States of the C_70_^**+**^ Cation

**DOI:** 10.1021/acsearthspacechem.5c00217

**Published:** 2025-10-17

**Authors:** Lisa Ganner, Gustavo A. Garcia, Martin Schwell, Miriam Kappe, Laurent Nahon, Elisabeth Gruber, Helgi Rafn Hrodmarsson

**Affiliations:** † Institute for Ion Physics and Applied Physics, 27255 University of Innsbruck, Innsbruck a-6020, Austria; ‡ Synchrotron SOLEIL L’orme des Merisiers Départementale, 128 91190 Saint Aubin, France; § LISA UMR 7583 Université Paris-Est Créteil and Université de Paris, 27010 Institut Pierre Et Simon Laplace, 61 Avenue du Général de Gaulle, Créteil 94010, France

## Abstract

The nature of the photoionization of fullerenes is of significant interest to molecular astrophysics and astrochemistry. The C_60_^+^ cation has been identified as a carrier of five of the diffuse interstellar bands (DIBs), and recent correlations between C_70_^+^ electronic bands and a few weak DIBs have been presented. In this work, we present a high-resolution electronic spectrum of C_70_^+^ recorded with He-tagging messenger spectroscopy, as well as the first threshold photoelectron spectrum (TPES) of C_70_. We comment on the He cage stability around C_70_^+^ and how it differs from that around other fullerenes, and we suggest some tentative vibrational assignments to the electronic spectrum based on a Jahn–Teller type formalism, which is expected from the C_70_^+^ system. We use a novel semiempirical method employing the high-resolution He-tagging spectrum and create band fits that we compare with the TPES to derive the adiabatic ionization energy of C_70_ (7.429 eV ± 0.015 meV). This methodology comes with some significant limitations but allows us to tentatively derive the energies of other excited states of the C_70_^+^ cation from the TPES.

## Graphical abstract

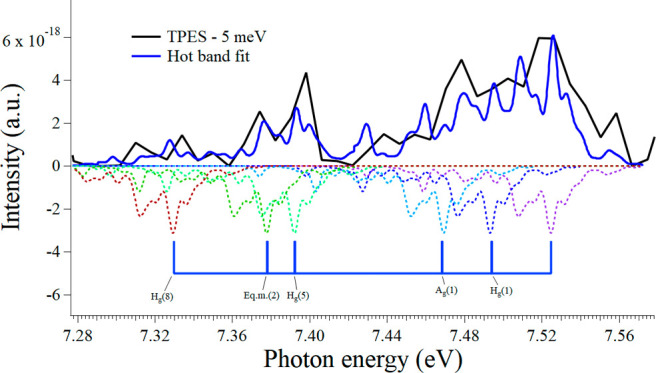

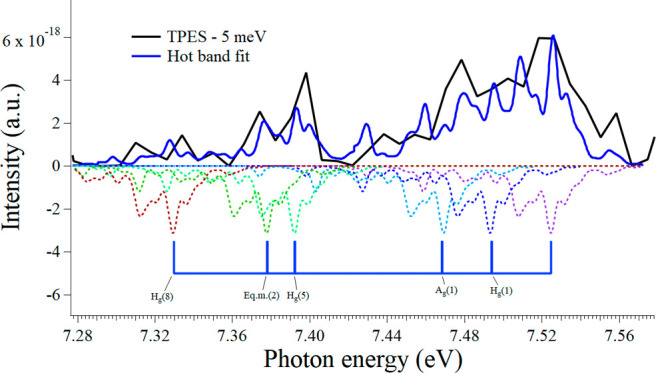

## Introduction

Fullerenes comprise an important component of interstellar matter. Since the detection of C_60_ and C_70_ in the Tc 1 planetary nebula (PN)^1^ and the identification of the C_60_^+^ cation as the first known carrier of the diffuse interstellar bands (DIBs),^2–6^ efforts have been devoted to spectroscopically investigating C_70_^+^ as a potential DIB carrier,^3^ and recent correlation analyses show some evidence to support the claim that the 7470.38, 7558.44, and 7581.47 Å DIBs could be assigned to C_70_^+^.^7^ Toward the Tc 1 PN, C_60_ and C_70_ are estimated to account for roughly 1% of the cosmic carbon budget.^1,8^ Although the original C_60_ and C_70_ discovery in Tc 1 seemed to favor the formation of fullerenes in C-rich PNe that are H-poor, they have now been observed in a variety of environments across the evolutionary cycle, from postasymptotic giant branch (AGB) type stars^9^ to proto-planetary nebulae (PPNe)^11^ as well as PNe.^12^ Interstellar fullerenes have made up an active field of research in astrochemistry^13^ which relates to explaining their presence and behaviors in terms of DIBs,^14^ their potential contribution to the 21 μm emission in PPNe,^15^ their formation starting from polycyclic aromatic hydrocarbons (PAHs)^16^ or from heating and energetically processing silicon carbide (SiC) presolar grains,^17,18^ and their contribution to the photoelectric heating of the interstellar medium (ISM).^19^

C_70_ shares an important property with C_60_, namely that of stability or robustness toward photodissociation, as its photodissociation energy exceeds its photoionization energy, i.e., 9.7 eV vs 7.4 eV.^20^ This robustness means that fullerene cations can be important drivers of exotic chemistry in the ISM, as has been shown through a variety of reactions.^21^ Besides its photostability, multiphoton ionization studies of C_70_ have revealed some peculiar aspects of its excited state and ionization dynamics, such as evidence of thermionic electron emission from vibrationally excited molecules.^22–25^ Additionally, C_70_ can form so-called superatom molecular orbitals (SAMOs) akin to C_60_,^26,27^ where excited electronic states of fullerenes can manifest as diffuse “hydrogen-esque” states with the electron density mostly localized in the center of the hollow carbon cage.

There have been several experimental^28–35^ and theoretical^36–41^ studies devoted to the ionization and electronic structure of C_70_. The photoelectron spectra in the gas phase have been investigated at different photon energies and have shown oscillations in the relative partial cross sections of the highest occupied molecular orbital (HOMO) and HOMO-1, attributed to the reflection of delocalized orbitals off the ball-shaped molecular potential.^42,43^ In addition, comparison of the gas-phase and solid C_70_ film spectra has helped elucidate spectroscopic and electronic properties of interest to advances in nanotechnology.^44,45^ Work has also been devoted to endohedral C_70_ ^46^ and C_70_ adsorbed on Cu^47^ and Ag^48^ surfaces, where charge transfer is observed from the metal surface onto the C_70_ cage. The IR spectrum of protonated C_70_ has also been recorded.^49^

The first successful electronic spectroscopic study on C_70_^+^ was a vibrationally resolved electronic absorption spectrum isolated in a 5 K neon matrix.^50^ Therein, the absorption of C_70_^+^ in the 12400–14000 cm^–1^ range was assigned to the E1′2←E1″2 transition which was guided by the first He I photoelectron spectrum of C_70_.^29^ Now, 35 years after the initial spectroscopic boom of the fullerenes, we present high-resolution electronic spectra of C_70_^+^ and subsequently use it to guide assignments of electronic states in the first threshold photoelectron spectrum (TPES) of C_70_.

With the combined advancements in He nanodroplet technologies and VUV synchrotron radiation coupled to double imaging photoelectron photoion coincidence (i^2^PEPICO) spectroscopy, we provide new high-resolution spectra and tentative vibrationally resolved assignments of the E1′2 excited state, leading to an estimated adiabatic ionization energy, as well as tentative assignments of the first few excited states of the C_70_^+^ cation in the TPES of C_70_, guided by the high-resolution electronic spectrum of C_70_^+^ recorded using He nanodroplet technology.

## Methods

### Experiment: Innsbruck

The experimental setup used to record the high-resolution electronic spectrum of C_70_^+^ in the region 12500–13700 cm^–1^ has been described in detail elsewhere.^51^ Hence, what follows is a brief description of the experiment. A supersonic expansion of helium gas was produced at roughly 8.5 K and 22 bar through a 5 μm-sized nozzle into a vacuum chamber. This allowed superfluid helium nanodroplets (HNDs) to form in the vicinity of an electron beam, producing positively charged HNDs in various charge states.^52^ These were guided through a differentially pumped chamber equipped with a spherical electrostatic analyzer. Afterward, the HNDs were guided into the pickup chamber, where the HNDs were doped with C_70_ which was evaporated from an ohmically heated oven. The picked-up C_70_ molecules were ionized by charge transfer or Penning ionization from cationic or metastable helium, respectively. After being doped, the HNDs entered the evaporation chamber, where the droplets collided with room-temperature helium gas, leading to helium evaporation from the droplet. Eventually, the gradually increasing Coulomb repulsion between the approaching C_70_^+^ ions resulted in the extraction of C_70_^+^ ions from the droplet. These ions can be tagged with a few helium atoms, the number of which can be steered by the pressure of the room-temperature helium gas in the evaporation chamber. Of these ions, C_70_He_2_^+^ and C_70_He_3_^+^ ions were selected with a quadrupole mass filter and merged with the beam of a pulsed, tunable laser (EKSPLA NT262), which was operated at 5 kHz. Absorption of a photon by the selected C_70_He_2/3_^+^ ions led to evaporation of the attached helium atoms. The formed bare C_70_^+^ ions were monitored with a time-of-flight mass spectrometer (TOF-MS) as a function of the photon energy, yielding the absorption spectrum. The spectra were corrected for the laser power fluctuations, assuming a linear dependence between the laser power and the ion counts. The laser wavelength was monitored and calibrated with a wavemeter (SHR high-resolution wide-range spectrometer). The line width of the EKSPLA NT262 laser is <3 cm^–1^ in the measured spectral range.

### Experiment: SOLEIL

The experimental details of the work at SOLEIL are similar to those described in previous work involving C_60_.^53,54^ At the DESIRS VUV beamline,^55^ we used horizontally polarized radiation in the range of 7.2–8.6 eV. The photon beam was filtered for high harmonics and dispersed by a 6.65 m normal incidence monochromator before entering the double imaging photoelectron photoion coincidence (i^2^PEPICO) spectrometer DELICIOUS3 ^56^ on the permanent endstation SAPHIRS, whose source chamber is separated from the spectrometer’s via a two-stage differential pumping scheme.^57^ A home-built stainless-steel oven was mounted inside SAPHIRS, where C_70_ and C_60_ were sublimated at 600 °C to generate sufficient vapor pressure. SF_6_ was used as the carrier gas with a backing pressure of 0.5 bar. SF_6_ was chosen as a carrier gas for its large mass in an effort to enhance vibrational cooling in the molecular beam, which Ar was unable to accomplish in previous work on C_60_.^53,54^

It has been previously suggested that the enormous size of C_60_ in comparison to Ar causes a significant velocity slip effect to occur because of the differences in mass.^58^ Another effect that can contribute is that there are not enough collisions to carry away the vibrational energy. The fullerenes’ reluctance to vibrationally cool could also be affected by their lowest vibrational modes (around 270 cm^–1^) being significantly higher than the lowest vibrational modes of smaller molecules like PAHs. To cool the fullerenes to their vibrational ground states, energy must be transferred from the lowest vibrational mode into translational energy, and the efficiency of this transfer decreases as the energy gap increases, thus making it more difficult for modes with higher energy to be completely cooled.

The supersonic expansion was created via a 500 μm nozzle and passed through two skimmers with a 2 mm orifice prior to entering the spectrometer (ionization) chamber. The molecular and photon beams crossed at the center of the spectrometer, and the resulting ions and electrons were, respectively, analyzed with a modified Wiley–McLaren mass spectrometer and a velocity map imaging device and correlated in time. The correlation scheme led to mass-selected photoelectron images for *m*/*z* 840, which were subsequently Abel-inverted using the pBasex algorithm^59^ to yield photoelectron spectra at each photon energy. The *m*/*z* 840 (C_70_ parent ion) signal was then obtained in matrix form and presented in Figure S1 as a function of the electron kinetic energy (*eKE*) and the photon energy, from which the TPES was extracted.^60^ This matrix could in theory be used to estimate the contribution to the photoelectric heating of C_70_ in the ISM, as we have done previously for PAHs;^61^ however, this comes with obvious limitations, as the maximum kinetic energy release of the photoelectrons here does not exceed 1.4 eV. The photon energy is calibrated using the 0–0 transition of C_60_ (7.598 eV)^54^ from its TPES, recorded simultaneously as a spectral calibrant.

## Results and Discussion

### Electronic Structure of C_70_^+^

Neutral C_70_ possesses D_5h_ symmetry, and therein, the HOMO is of *a*_2_″ symmetry, with an *e*_1_″ HOMO-1 very close in energy.^37,40^ Based on early calculations, along with the first photoelectron spectrum,^29^ it was suggested that the ground state was *E*_1_″ symmetric, which would account for several low-energy transitions observed in the photoelectron spectrum.^50^ It is useful here to interject that ′ and ″ denote symmetric and antisymmetric orbitals (or stretches) with respect to the symmetry plane, respectively.

With an *e*_1_″ ground state, the C_70_^+^ cation could access several low-lying excited states, namely, E2″2←E1″2 , E1′2←E1″2 , E1′2←E1″2 , E2″2←E1″2 , predicted at 5200, 7700, 12700, and 14600 cm^–1^, respectively. Additionally, there would be two forbidden transitions at lower energies, namely A2′2←E1″2 and E2′2←E1″2 . Hence, Fulara et al.^50^ deduced that since the allowed transitions from the ground state, assuming A2″2 symmetry, were outside the energy range where several convoluted features were observed in the photoelectron spectrum,^29^ the C_70_^+^ cation should be E1″2 symmetric. This suggests that upon losing an electron, the *a*_2_″ and *e*_1_″ symmetric MOs energetic ordering switches.

Perhaps surprisingly, there has been limited research dedicated to the electronic structure of the C_70_^+^ cation. In a recent computational study of the microwave spectrum of C_70_^+^, Nemes^62^ computed that the HOMO is likely a result of orbital mixing involving the doubly degenerate *e*_1_″ and singly degenerate *a*_2_″ orbitals (Figure 1a,b), which is also consistent with prior calculations by Zakrzewski et al.^40^

**Figure 1:**
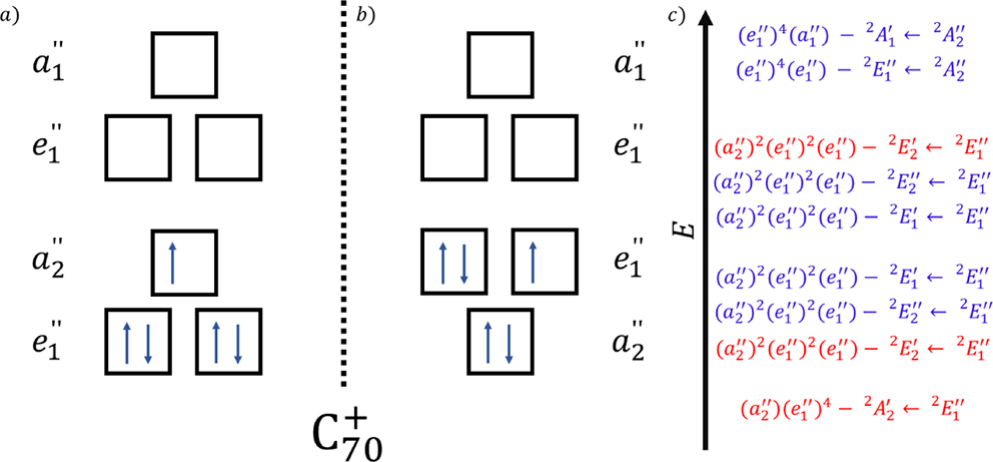
Panels (a,b) show the electronic configuration of the ground state of C_70_^+^ as it likely comprises two accidentally degenerate configurations according to Nemes.^62^ Panel (c) shows the configurations of the possible electronically excited states of C_70_^+^ as described in the work of Fulara et al.^50^ Red color is used for forbidden transitions, and blue is used for allowed transitions. However, the mixed nature of the ground state possibly allows forbidden transitions.

In Figure 1c, the corresponding configurations of the excited states formed by allowed (in blue) and forbidden (in red) transitions starting either from E1″2 or A2″2 symmetric ground states are shown in the energetic order they were expected in the work of Fulara et al.^50^ While this is solid reasoning, in light of the doubly degenerate *e*_1_″ and singly degenerate *a*_2_″ orbitals being accidentally degenerate, we can expect that the ground state is effectively “softened” by manifesting pseudo-Jahn–Teller effects.^63^ This can be seen in the electronic configuration of the forbidden A2′2←E1″2 transition, or $\left(a_{2}^{\prime \prime }\right){\left(e_{1}^{\prime \prime }\right)}^{4}$ , which mimics the electronic configuration of the A2″2 symmetric ground state. Likewise, the same initial forbidden transition starting from the A2″2 symmetric ground state (i.e. E1″2←A2″2 ) would give ${\left(e_{1}^{\prime \prime }\right)}^{3}{\left(a_{2}^{\prime \prime }\right)}^{2}$ , which mimics the configuration of the E1″2 symmetric ground state.

This mixed nature of the ground state of C_70_^+^ is reminiscent of the ground state of C_60_^+^ which has two low-lying electronic states that were theorized to be dark but have since been observed experimentally.^54,64^ These excited states also happen to “soften” the ground state, allowing excitations outside of its Franck–Condon region. This type of pseudo-Jahn–Teller (JT) effect has been theorized as the reason for the sharp bands from C_60_^+^ electronic excitations in the DIB transitions.^54,64,65^ However, whereas in C_60_^+^, the D_5d_ ground state symmetry breaking is cushioned by the C_2h_ symmetry retained in the excited state, in C_70_^+^, the cation’s symmetry is already reduced to C_S_ symmetry from the D_5h_ symmetry of neutral C_70_.^62^ As such, the greater breaking in symmetry of C_70_^+^ means that the vibrational profile of the molecule becomes more complex in comparison to its neutral C_70_ counterpart.^66,67^ Hence, we can expect C_70_^+^ to showcase convolved JT active bands whose individual energies and intensities will subtly differ depending on the electronic configuration. As Nemes also points out in previous work,^68^ the symmetry of the mixed ground state involves the direct product of the D_5h_ species *e*_1_″ and *a*_2_″ which should yield *e*_1_′ in a pseudo-JT interaction scheme.

These particular JT effects have not been studied theoretically for the C_70_^+^ system, but we can draw some direct comparisons to the work of Tian et al.^69^ who studied the JT splitting of C_70_^3–^. If we assume the ground state of C_70_^+^ has *e*_1_″ symmetry and an *a*_2_″ orbital very close in energy, then the configuration of C_70_^3–^ is practically the same, but instead involves an *a*_1_″ orbital very close in energy. Likewise, C_70_^+^ involves electron filling in degenerate orbitals, which also applies to C_70_^3–^. Tian et al. computed 12 *a*_1_″, 21 *e*_2_′, and 22 *e*_1_′ symmetric JT-active bands whose energies range from 187 to 1738 cm^–1^. Hence, for each electronic state of C_70_^+^, we can expect a convolution of up to 50 different vibrational bands, as well as their overtones and combination bands.

### Electronic Spectrum of C_70_^+^

The recorded electronic spectrum of C_70_^+^ is shown in Figure 2 along with comparisons to the Gaussian fits to the recorded spectrum by Campbell et al.,^3^ who obtained their experimental spectrum by He-tagging messenger spectroscopy in a cryogenic ion trap. For the sake of clarity, we opt to present their Gaussian-fitted spectrum. In Figure 2, we also present the spectrum of Fulara et al.,^50^ which was recorded in a frozen Ne matrix. The Fulara spectrum was shifted by 20 cm^–1^ to account for the spectral shift induced by the matrix. The frozen Ne matrix also significantly broadened the peaks, which made assignments difficult but not impossible in the work of Fulara et al. The digitized fitted spectrum of Campbell et al. compares well with our recorded spectrum, but we were able to decipher several more peaks.

**Figure 2:**
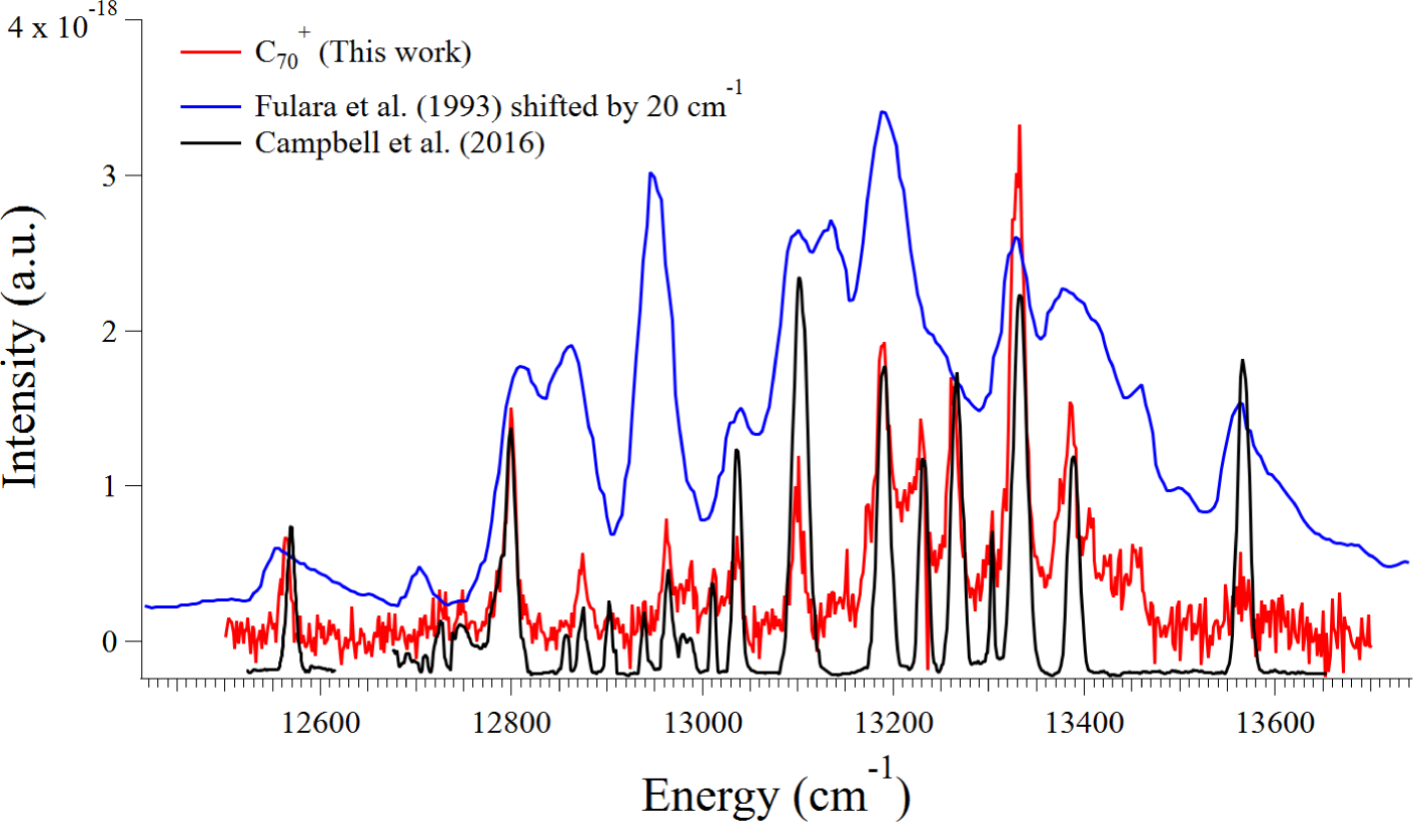
Our recorded electronic spectrum of C_70_^+^ is shown in red. The Fulara et al.^50^ spectrum is shown in blue. In black, we show the Gaussian fits that Campbell et al. applied to their recorded spectrum.^3^

In Figure 3, we present a more direct comparison between our spectrum and the spectrum of Campbell et al.,^3^ where we provide identification of the additional peaks that we can resolve. The peak positions are presented in Table 1 along with the band energies calculated by subtracting the observed peak position from the 0–0 transition. We also provide comparisons to the work of Campbell et al.^3^

**Figure 3:**
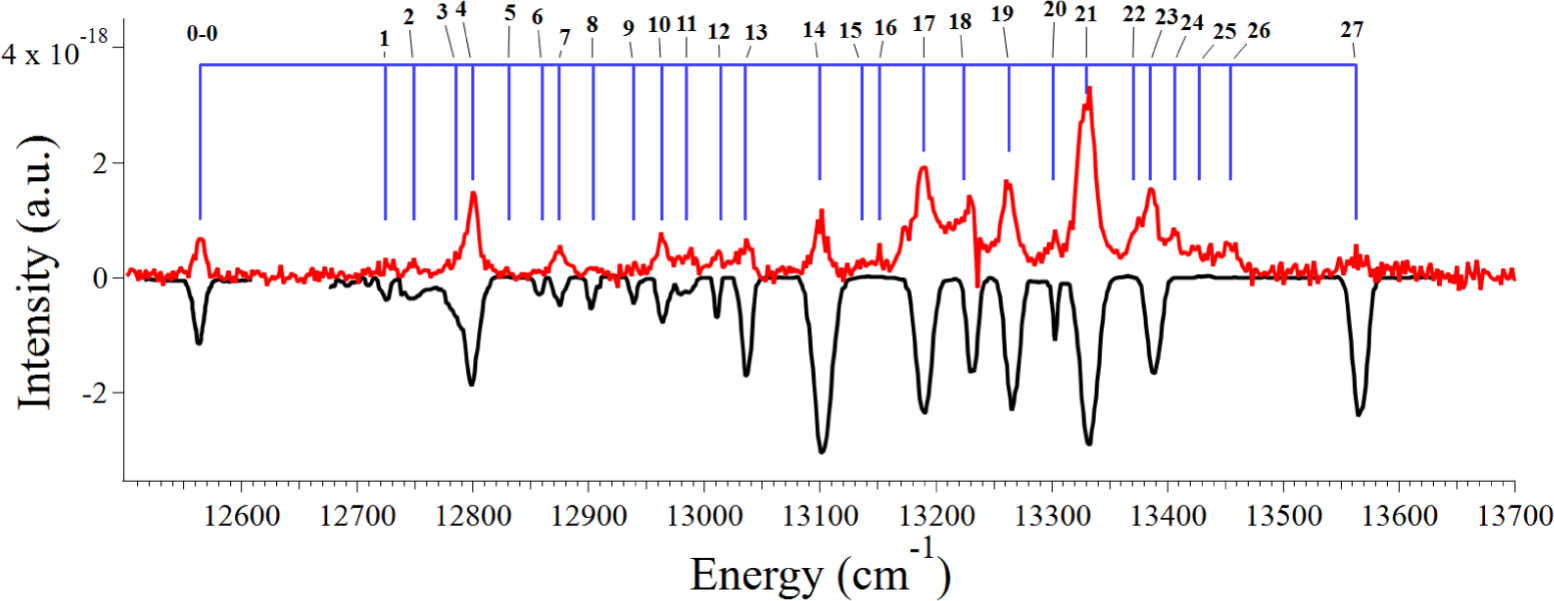
Our recorded spectrum is shown in red. The Gaussian-reduced spectrum of Campbell et al.^3^ is inverted and shown in black. Shown in blue is an assignment bar identifying visible peaks, which are tabulated in Table 1. Potential assignments to each numbered peak are presented in Table S1.

**Table 1:** Peak Positions and Band Energies (Δ) along with Comparisons to the Work of Campbell et al^3^

Peak number	Cation (This work)	Δ	Cation (Campbell)	Δ
	12564.0	0	12564.1	0
1	12727.2	163.2	12726.5	162.4
2	12747.7	183.7	12750.1	186.0
3	12785.0	220.8	12785.6	221.5
4	12799.3	235.3	12801.0	236.9
5	12831.0	267.0	-	-
6	12856.7	292.7	12858.1	294
7	12874.7	310.7	12876.0	311.9
8	12904.1	340.1	12903.2	339.1
9	12939.0	375	12939.8	375.7
10	12962.9	398.9	12964.6	400.5
11	12984.6	420.6	12983.8	419.7
12	13012.5	448.5	13011.0	446.9
13	13035.3	471.3	13036.6	472.5
14	13099.7	535.7	13101.7	537.6
15	13136.0	572	-	-
16	13152.4	588.4	-	-
17	13189.3	625.3	13188.6	624.5
18	13223.8	659.8	13230.3	666.2
19	13262.8	698.8	13264.9	700.8
20	13300.9	736.9	13301.4	737.3
21	13330.4	766.4	13330.5	766.4
22	13370.8	806.8	-	-
23	13386.2	822.2	13386.5	822.4
24	13406.2	842.2	-	-
25	13427.4	863.6	-	-
26	13454.6	890.6	-	-
27	13562.7	998.7	13563.4	999.3

The assignments made by Fulara et al.^50^ were guided in part by calculations of active vibrations in the neutral ground state of C_70_ by Procacci et al.^70^ C_70_ has 122 vibrational modes $(12A_{1}^{\prime }+9A_{2}^{\prime }+21E_{1}^{\prime }+22E_{2}^{\prime }+9A_{1}^{\prime \prime }+10A_{2}^{\prime \prime }+19E_{1}^{\prime \prime }+20E_{2}^{\prime \prime }).$ The 12 totally symmetric $A_{1}^{\prime }$ vibrations are highly polarized and Raman active. They were used by Procacci et al. to scale and benchmark the frequencies to compare with available experimental data. The 12 $A_{1}^{\prime }$ fundamental vibrations of C_70_ have frequencies between approximately 270 and 1560 cm^–1^, and these were used as the principal guide in the assignments of Fulara et al.^50^ There were, however, several other peaks that were tentatively assigned as JT active vibrations, and matrix effects were cited as potential originators of symmetry lowering in the excited state. In the case of C_60_^+^, Lykhin et al.^65^ predicted a splitting on the order of 1–16 cm^–1^ for the addition of a He atom to the fullerene cage. Here, for the addition of 2–3 He atoms to C_70_^+^, such splitting should be observable, and they could, in principle, contribute to the broadening of the peaks. However, this is not immediately evident. For the time being, we will assume that matrix effects are bypassed in this work.

In the Supporting Information, we present another version of Table 1, where tentative assignments are provided for the identified peaks. By using the computed frequencies of Tian et al.^69^ on C_70_^3–^ to guide our assignments, we can deduce that the spectral region contains a wealth of overtones and combination bands, and identifying a single fundamental frequency is exceedingly difficult. The complete inclusion of the multiple bands anticipated in a JT-active system as complex as C_70_^3–^ or C_70_^+^ can only allow tentative assignments to the multiple features observed in the spectrum, but the assignments quickly become somewhat uncertain due to the large number of potential overtones and combination bands.

These assignments differ significantly from the work of Fulara et al.,^50^ whose spectrum suffered from significant broadening and shifts due to the Ne matrix. Second, their assignments were guided by comparisons to the electronic spectrum of neutral C_70_. This provided adequate comparisons (errors ranging from 5 to 80 cm^–1^), but it also left out multiple other bands that have since been resolved—first in the work of Campbell et al.^3^ and now in this work. One way of obtaining deeper insights into the nature of the activated bands is to investigate the stability of the He cage around the strongest peaks.

### He Cage Stability around C_70_^+^

In Figure 4a, the central positions of three major absorption bands of C_70_He_*n*_^+^ (labeled 19, 21, and 23 in Figure 3) as a function of the mean number of attached helium atoms *n* are shown. For comparison, the He-taggant dependency of the two electronic origin bands of C_60_He_*n*_^+^ is displayed in b. In the case of C_70_^+^, the absorption frequency of the three studied bands shows a clear minimum at 37 attached helium atoms, which corresponds to the sum of hexagonal (25) and pentagonal faces (12). This is analogous to the case of C_60_^+^, which has 20 hexagonal and 12 pentagonal faces, and where the minimum was reached at 32. These first 32 helium atoms for C_60_^+^ and 37 for C_70_^+^ are thought to form a solid layer around the ionic core.^71,72^ For *n* > 37, an almost linear blueshift follows the initial redshift up to about *n* = 62, where mass spectra indicate a shell closure.^71^ Analogous to C_60_^+^,^71,72^ this blueshift is likely associated with the formation of a liquid layer intermixed with a solid layer. Beyond *n* = 62, the matrix shift remains at about the same level, reflecting the transition to superfluid bulk helium.

**Figure 4:**
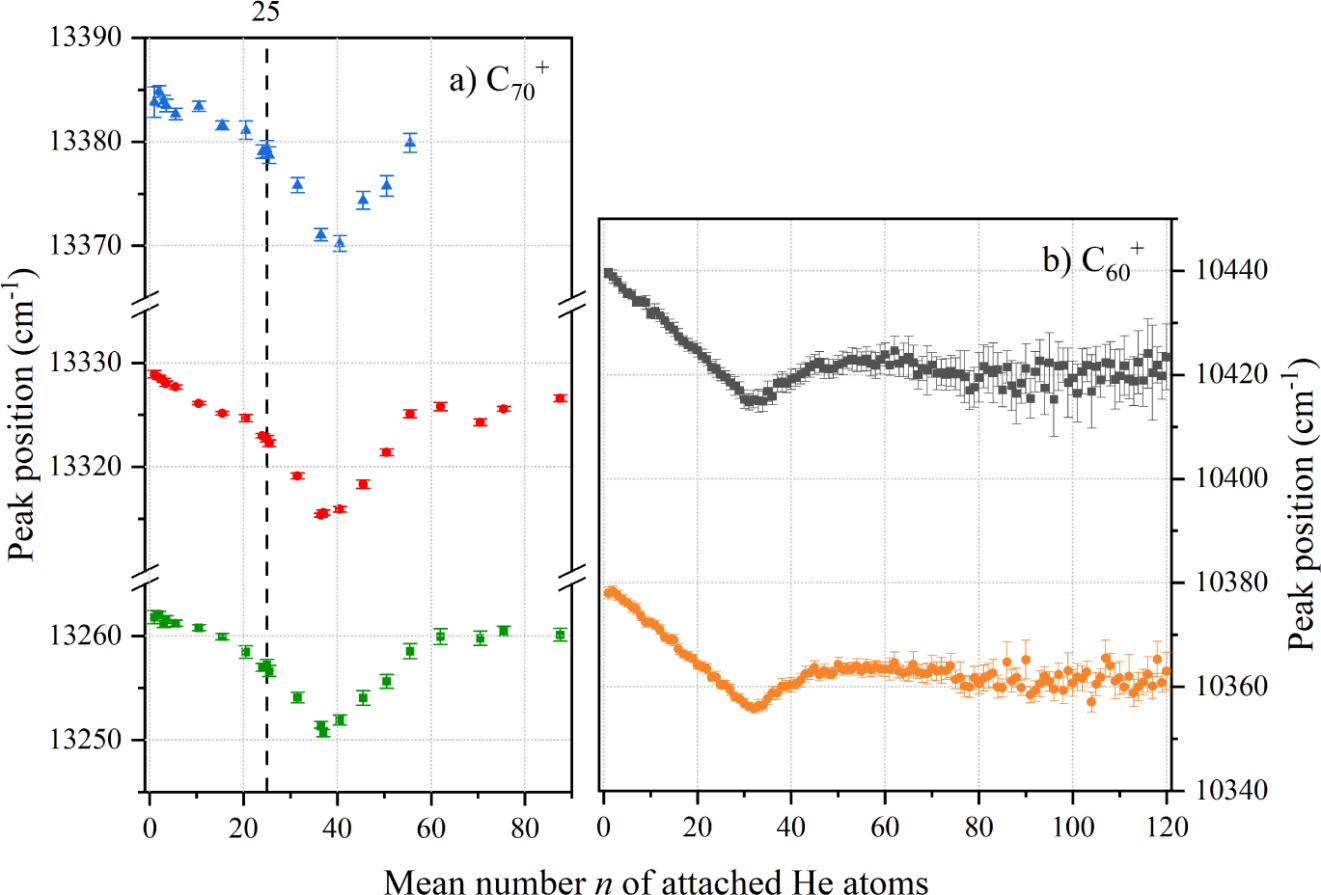
Central position of (a) three C_70_He_*n*_^+^ absorption bands which are labeled 19 (green), 21 (red), and 23 (blue) in Figure 3, and (b) the two electronic origin bands of C_60_He_*n*_^+^ in orange and gray as a function of the mean number of attached helium atoms, *n*. Parts of (b) have been published elsewhere.^73^

In comparison to the data for C_60_^+^, which follows a linear trend between 1 and 32 attached He atoms, the shift in C_70_^+^ shows a rather nonlinear dependency for the first 37 He atoms. A similar trend was found in the case of the C_60_ anion.^73^ While the reason for these differences is not clear, we propose two factors that could contribute to the observed behaviors.

In C_60_^+^, each of the first 32 He atoms is bound by roughly the same binding energy, and accordingly, each additional helium atom contributes the same amount of redshift.^71,72^ Prior calculations on C_60_^+^ by Leidlmair et al.^71^ indicated that the binding energy of helium above a hexagon is slightly larger (10.3 meV) than above a pentagon (9.0 meV); however, this difference in binding energy might be too small to be apparent in the seemingly perfectly linear helium matrix shift. In contrast to C_60_^+^, the binding energy of helium on the C_70_^+^ cage is expected to vary to a greater extent across the surface due to its lower symmetry. This could potentially explain the observed curvature in the helium-dependent absorption band position in Figure 4. One might even perceive a kink in the data at *n* = 25 He atoms, which could be tentatively attributed to a preferential occupation of the 25 hexagons, followed by the 12 pentagons. However, a similar nonlinear behavior was also observed in the case of C_60_^–^,^73^ so this might not be the only reason.

Another reason for this could lie in the nature of the transitions. The C_60_^+^ transitions which were studied as a function of the number of helium atoms are single bands of a single symmetry, namely due to the pseudo-JT splitting in the ^2^ *E*_g_ excited state of C_60_^+^.^65^ In contrast, as suggested by our tentative vibrational assignments in Table S1, the respective C_70_^+^ bands do not consist of a single unique vibrational mode but comprise a degeneracy of several bands of different symmetries, namely *a* and *e* symmetric vibrations as an E⊗e JT-active system.^69^ Hence, the band dependency for the first 37 He atoms could result from multiple bands of different symmetries contributing to the bands. That is, different symmetric vibrations favor the induced losses of some He atoms over others in an effort to conserve that particular symmetry. This would also explain the same trend observed in the C_60_ anion.^73^ In C_60_^–^, its JT-active modes are expected to be *h*_g_ symmetric according to theory.^74^ As the attached electron occupies the triply degenerate t_1u_ LUMO of C_60_, each *h*_g_ mode is split into three components. The degeneracy is lifted, leading to *L* = 1, 2, 3 orbitals of symmetry *T_1u_*, *H_u_*, and *T_2u_* ⊕̅*G_u_*. This could lead to three separate contributions to the He-tag-dependent shift of the absorption bands in C_60_^–^.

For a definitive conclusion, this requires further investigation, for instance, utilizing molecular dynamics simulations and detailed ab initio computations, which are beyond the scope of this work.

### Photoionization of C_70_: 0–0 Transition and Making a Model Spectrum

Our previous work involving the assignments of the TPES of C_60_ was made significantly more difficult by the high temperature used in the experiment. The high temperatures activated multiple hot bands, which made assigning the 0–0 transition in C_60_^+^ a difficult practice that involved careful analysis of a few TPES at different maximum electron kinetic energy (*eKE*_max_) values.^53,54^ For the experiment involving C_70_, we opted to use a heavier carrier gas, SF_6_, in an effort to bypass or at least reduce the appearance of hot bands that would complicate the analysis. The mass of SF_6_ is 3.65× greater than that of Ar, which was previously used, and in theory, this should more effectively cool the vibrationally hot molecules in the molecular beam. However, whereas C_60_^+^ encompasses a reasonably straightforward *H*_g_ vibrational mode progression due to the dynamic JT effect in the ground state,^75^ the vibrational profile of C_70_^+^ is more complex than that of C_60_^+^ which nonetheless results in multiple hot band contributions that are important to characterize to successfully locate the 0–0 transition in C_70_^+^. Franck–Condon factors (FCFs) are important as well, and these are likely to be different starting from the neutral undistorted C_70_. However, these are not currently available, and the difficulties involved make such calculations out of scope for this manuscript. Thus, as a zeroth-order approximation, we shall use the electronic spectrum of C_70_^+^ described above to build a model of what transitions to expect from the neutral to the ground and excited states of C_70_^+^ in the TPES. This provides us with a semiempirical method for estimating the energies for the electronically excited states in C_70_^+^.

Accounting for hot bands in C_70_ is difficult due to the large number of vibrational modes (122, including 82 doubly degenerate).^70,76,77^ A restriction is needed a priori because of the enormity of the parameter space offered by the inclusion of all the C_70_ vibrational modes. Restricting the construction of the hot band fits to the $A_{1}^{\prime }$ symmetric modes allows us to sample contributions of hot bands over the expected energy region, from the lowest energy vibrations (around 250 cm^–1^) and the highest energy vibrations (close to 1600 cm^–1^). This also prevents overfitting but forces us to make some general assumptions regarding the primarily contributing hot bands.

Recently, we simulated hot bands in the second photoelectron band of C_60_ ^78^ and found that Raman-active bands provide significantly better fits compared to IR-active modes.^54^ C_70_ has 12 such Raman-active bands, which possess $A_{1}^{\prime }$ symmetry. Ten of them correlate with the Raman-active bands in C_60_ as described previously,^70^ where eight bands of *H*_g_ symmetry and two bands of *A*_g_ symmetry contribute. As C_70_ possesses ten more carbon atoms around the molecular equator, there are two additional equatorial modes (Eq. m.) present. We will use these labels in the following discussion. This approximation provides a necessary constraint to prevent overfitting, instead of fitting over 200 contributing modes to the hot bands.

To identify the ground state and its 0–0 transition (and thus the adiabatic ionization energy of C_70_) we devised the following procedure. The electronic spectrum was first smoothed using a smoothing algorithm in the Igor Pro software (https://www.wavemetrics.com) to reduce the resolution of the electronic spectrum so that the vibrational structure could be comparable to that observed in the TPES. We note that between 7.43 and 7.55 eV, the TPES shows spectral features roughly consistent with those found between 13150 and 13450 cm^–1^ in the electronic spectrum of C_70_^+^. However, the features in the electronic spectrum below 13150 cm^–1^ are not well characterized in the TPES due to the presence of hot bands, which hide the 0–0 transition. Thus, we assume that each hot band contributes the same photoelectron spectrum as a cold molecule would, only shifted by the energy of the Raman-active vibrations. Then, we simulated hot bands by coadding the electronic spectra that have been shifted by their Raman-active vibrational modes (see Figure 5a). Each coadded spectrum is fitted with a scaling factor such that all coadded shifted spectra best replicate the TPES. Figure 5a shows the resulting TPES obtained by setting the *eKE*_max_ value to 5 meV.

**Figure 5:**
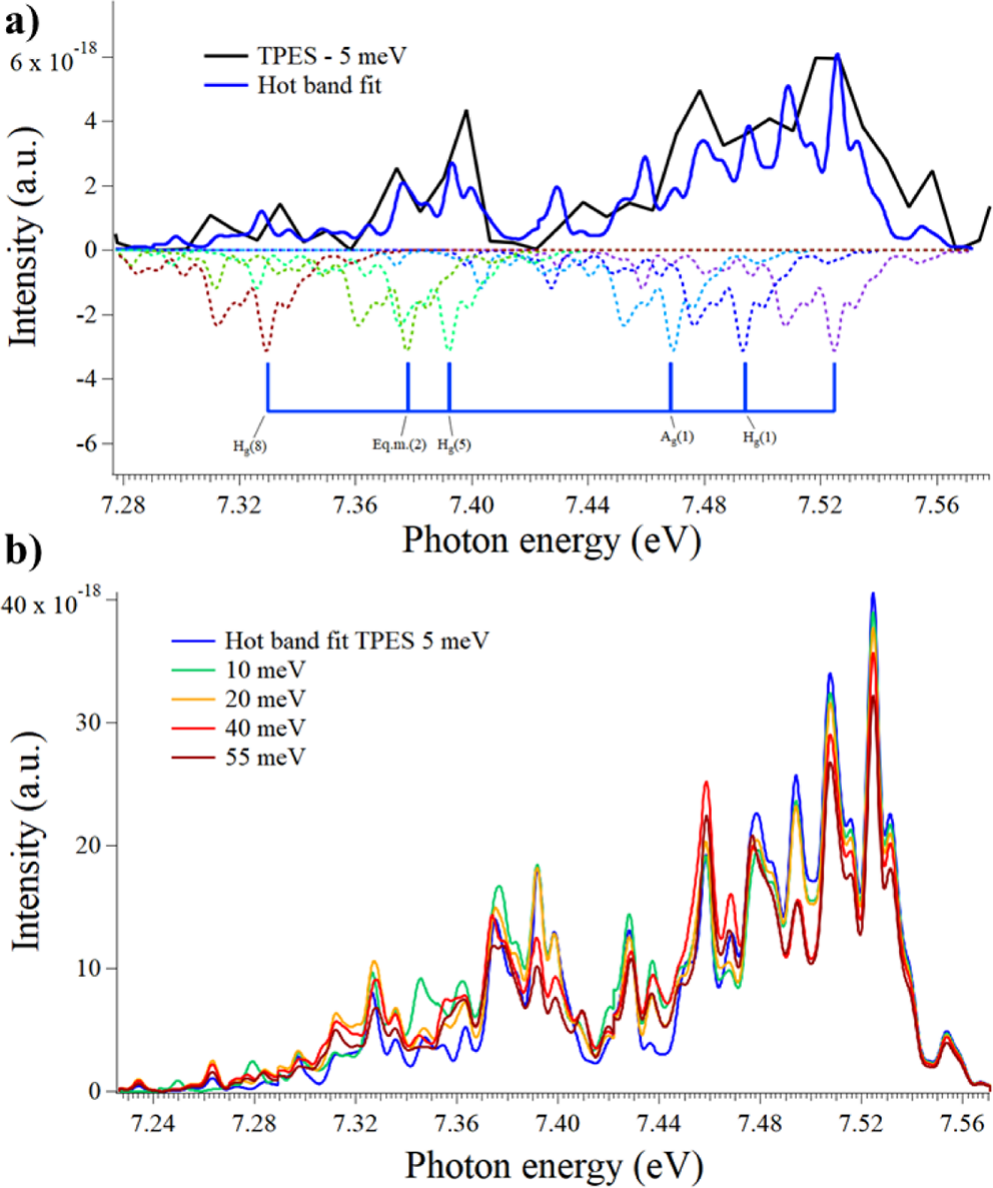
(a) Summation of coadded smoothed electronic spectra to replicate the ground state and associated hot bands in the 5 meV TPES. (b) All hot band fits derived to replicate the ground state in the different TPES.

This procedure was followed for a few iterations of the TPES which were created by setting *eKE*_max_ = 5, 10, 20, 40, and 55 meV. We did this to verify the consistency of the fitting method. This gave rise to the so-called hot band fits shown in Figure 5b where each one was created using the individual scaling factors used to simulate the different hot bands. These scaling factors are listed in Table 2.

**Table 2:** Hot Band Scaling Factors Used to Make the Hot Band Fits to Identify Excited States in the TPES

		Derived scaling factors from TPES with different *eKE*_max_				
Hot band symmetry	Hot band energy (cm^–1^)	5 meV	10 meV	20 meV	40 meV	55 meV
Fundamental	0	0.463 ± 0.008	0.412 ± 0.009	0.410 ± 0.007	0.405 ± 0.007	0.410 ± 0.008
*H*_g_(1)	253	0.231 ± 0.008	0.194 ± 0.009	0.194 ± 0.009	0.095 ± 0.009	0.115 ± 0.010
*H*_g_(2)	393	0	0	0.0267 ± 0.011	0.078 ± 0.012	0.129 ± 0.014
*A*_g_(1)	448	0.056 ± 0.008	0	0	0.056 ± 0.013	0.018 ± 0.016
Eq.m.(1)	564	0	0.059 ± 0.010	0.066 ± 0.007	0.078 ± 0.013	0.037 ± 0.014
*H*_g_(3)	702	0	0.042 ± 0.010	0	0	0
*H*_g_(4)	709	0	0	0	0.013 ± 0.009	0.031 ± 0.010
*H*_g_(5)	1061	0.203 ± 0.009	0.163 ± 0.010	0.182 ± 0.009	0.113 ± 0.009	0.096 ± 0.012
Eq.m.(2)	1185	0.013 ± 0.009	0.066 ± 0.010	0.027 ± 0.012	0.022 ± 0.013	0.051 ± 0.016
*H*_g_(6)	1229	0	0	0.020 ± 0.012	0.071 ± 0.013	0.057 ± 0.014
*H*_g_(7)	1450	0	0.064 ± 0.007	0	0	0
*A*_g_(2)	1472	0	0	0	0	0
*H*_g_(8)	1575	0.034 ± 0.008	0	0.075 ± 0.007	0.069 ± 0.007	0.055 ± 0.008

Once the hot band fits for the different TPES were created, we attempted to manually fit the hot band fits to the rest of the TPES in an effort to locate the different excited states. The result is shown in Figure 6 where the TPES has *eKE*_max_ = 40 meV. We also attempted to do this for the other iterations of the TPES with different *eKE*_max_ values (see Figure S2). This turned out to be exceedingly difficult due to the TPES being very complex, which poses challenges in accurately locating vibrational trends. These issues are exacerbated by the weak and noisy TPES signal. As can be seen in Figure 6, this correspondence is not perfect; however, the hot band fits capture recurring trends in the TPES reasonably well. It is important to clarify here that the TPES obtained starting from neutral C_70_ and the electronic absorption spectrum starting from C_70_^+^ will possess different Franck–Condon factors and different vibrational overlaps. Thus, the quality of the fit cannot be solely judged based on matching intensities but rather on matches in spectral positions.

**Figure 6:**
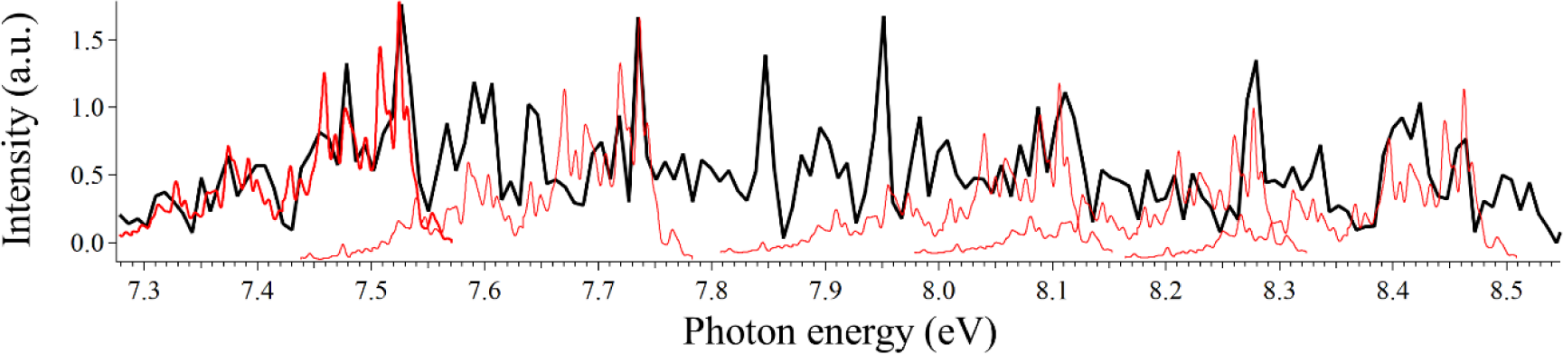
TPES of C_70_ obtained with *eKE*_max_ = 40 meV. The red bold lines trace the hot band fits created to fit the 0–0 transitions to the ground state. The thin-lined red traces are the best matches of the hot band fits to the TPES used to tentatively identify the excited states of the cation.

This semiempirical assignment methodology comes with several caveats. We collected the TPES starting from 7.23 eV, and there is an indication that some hot band features were missed, for example, in the leftmost part of the TPES. This could mean that peaks and intensities were lost in the making of the hot band fits, which could contribute to the lack of signal correspondence between 7.78 and 7.87 eV. We also assume that all excited states and the ground state of C_70_^+^ possess the same vibrational structure (or the same fundamental vibrations and resulting vibrational profile) as the E1′2 state shown in Figure 2 and 3. As has been observed in differences between excited states of neutral and cationic C_70_,^50^ there may be some slight differences among their frequencies, especially given the innate complexity of the vibrational patterns that plague the JT-active states of C_70_ in different charge states (see above). Different hot bands may also play different roles in different excited states. We can thus expect some slight peak shifts in our comparisons, on top of the inconsistent intensities expected from comparisons of a TPES and an electronic absorption spectrum.

There are also caveats concerning the fitted coefficients presented in Table 2. The different hot bands do not follow a perfect Boltzmann distribution. Figure 7 shows the expected values for the normalized scaling factors, assuming Boltzmann distributions at temperatures from 100 to 1500 K. While hot bands below 1000 cm^–1^ appear to follow a Boltzmann-esque distribution corresponding to temperatures between 100 and 500 K (average 370 ± 100 K), above 1000 cm^–1^, the distributions correspond to temperatures above 500 K. In the case of the *H*_g_(5) band, its highest temperature estimate is as high as 1800 K. This is surely an outlier, as the oven temperature in the experiment was set to 873 K. There are some Raman-active bands (see Table 6 of Schettino et al.^77^) belonging to different symmetries than the *H*_g_(5) band around 1000 cm^–1^, and their inclusion might lower the scaling factor for the involvement of the *H*_g_(5) band. However, further increasing the number of active bands contributing to the hot bands also leads to additional issues of overfitting, which we have tried to limit for consistency.

**Figure 7:**
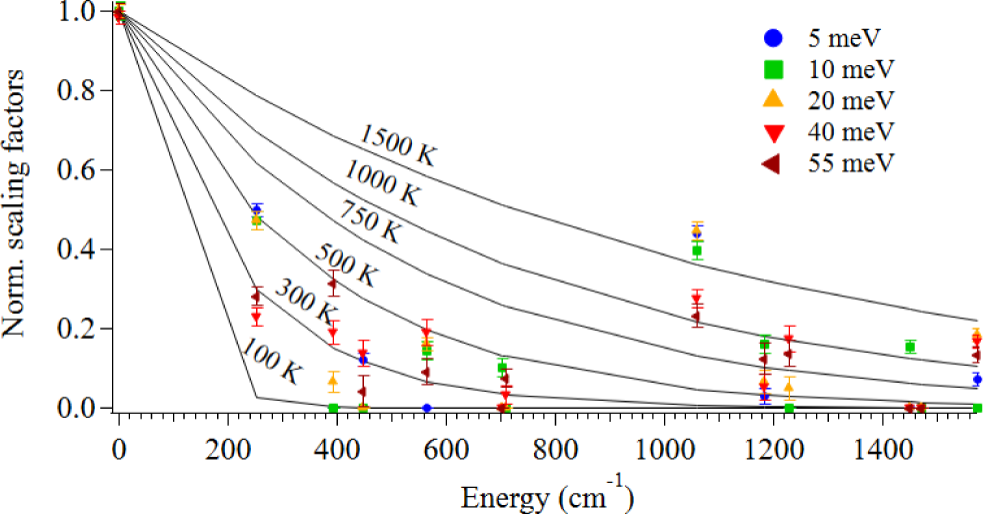
Scaling factors derived from the hot band fits in Table 2 were normalized here and plotted in blue (5 meV), green (10 meV), orange (20 meV), red (40 meV), and brown (55 meV), along with the expected Boltzmann behavior at different temperature regimes depicted as black lines.

Treating the *H*_g_(5) band as an outlier, the average temperature from the fitted bands above 1000 cm^–1^ corresponds to 900 ± 270 K, which is close to the oven temperature. However, the average temperature from all the normalized scaling factors in Table 2 gives an average temperature of 720 ± 460 K. Ignoring the large uncertainty, this is more or less the same value as the translational temperature that we calculated for C_60_, or 735 K, from the width of its velocity distribution in our previous work.^54^ This applies to the distributions that contributed to the hot bands, as many of them were not found to contribute in any meaningful way and were thus assigned zero values.

It is also worth noting that a non-Boltzmann distribution is not a priori surprising. The vibrational relaxation of large molecules is also known to depend on the vibrational state. For instance, in pyridine, some vibrational states relax so much that they are not observed, while others do not relax at all.^79^ Additionally, these effects are dependent on the type of carrier gas.

Although these inconsistencies in the Boltzmann distribution show the inherent limitations of estimating the hot bands as being derived from the Raman-active bands of a single symmetry in neutral C_70_, it is worth adding that the experimental conditions are fairly complex themselves. The hot C_70_ molecules embedded via collisions in a supersonic beam of SF_6_ will lead to a mixed population ensemble of cooled and not-so-cooled molecules of both species. Likewise, since C_60_ was also in the mix as a spectral calibrant, this might contribute to the non-Boltzmann behavior of the hot band scaling factors.

Considering these caveats, we can provide only tentative assignments to the energies of the excited states. Nonetheless, they agree well with those of Lichtenberger et al.,^29^ who conducted the first measurement of the photoelectron spectrum of C_70_ (see Table S2). We derive the 0–0 transitions from the TPES by comparing it with the hot band-fitted spectra and the electronic spectra in Figure 5a,b. For example, the 0–0 transition in the ground state should appear approximately 766 cm^–1^ below the strongest peak (no. 21) in the series of peaks labeled no. 17–24 in Table 1. This gives the adiabatic ionization energy as 7.429 eV ± 0.015 meV.

There are several factors required to estimate the uncertainty of the assignments in Table S2. First, there is the accuracy of the calibration. We used the 0–0 transition of C_60_ which was previously measured with a 5 meV accuracy under almost the same experimental conditions.^54^ However, convolution of the photon energy resolution (3 meV) and the electron bandwidth used to create the TPES (*eKE*_max_ = 40 meV) should yield a total energy resolution of around 13 meV. The combination of errors resulting from the signal-to-background ratio and the total energy resolution will give us an uncertainty on the order of 15 meV. One final note on the uncertainty concerns the anticipated frequency shifts due to the increased temperature. Both IR- and Raman-active vibrations in fullerenes are known to shift with increasing temperatures.^80–82^ These can induce shifts of around 3–5 meV for temperatures between 700 and 1000 K.^54^

## Conclusion

In this work, we present the electronic spectrum of C_70_^+^ recorded using He-tagging messenger spectroscopy. It generally compares well with the previous spectrum of Campbell et al.,^3^ but we can identify a greater number of bands in our spectrum. We also show how the He cage stability around C_70_^+^ differs from that of C_60_^+^ and is more reminiscent of that of C_60_^–^. We suggest that the cage stabilities are affected by a combination of factors, namely the nature of the He cage surrounding the fullerene and the activated vibrational modes in the fullerenes when the resonances of the fullerenes embedded in the He cage are activated. In the case of C_70_^+^, this involves a complicated manifold of JT-active bands whose proper theoretical treatment is outside the scope of this article.

We also present the first TPES of C_70_ which turns out to be exceedingly complex due to a combination of two factors, namely, the complex vibrational structures of the different ionic states and the temperature of the experiment, leading to a convolution of hot bands contributing to the TPES. In previous work on C_60_, we used a combination of molecular dynamics simulations to establish the contribution of many conformers to the TPES and a previously computed ab initio spectrum and Franck Condon simulation to establish the contribution of a single conformer to the TPES. Here, we attempt an original procedure that uses the recorded high-resolution electronic spectrum, albeit with limitations since the initial state is not the same, which yields a satisfactory agreement with the TPES but with multiple caveats that disfavor concrete assignments of the TPES. However, this still gives us the means to estimate the adiabatic ionization energy as 7.429 eV ± 0.015 meV, as well as the energies of the first five outer-lying states of the C_70_^+^ cation.

While the fitting methodology presented in this paper comes with several obvious caveats, it should be noted that more rigorous theoretical treatments could be viable. For instance, quasi-diabatic models of the Hamiltonian that correctly describe the physics for many small JT and pseudo-JT active molecules could be used.^83,84^ However, the high dimensionality of the C_70_ fullerene would be an obvious challenge for which vibronic dynamical methods like MCTDH might be suitable.^85^

## Supplementary Material



## Notes

It provides the recorded two-dimensional photoelectron spectrum matrix of C_70_ between 7.36 and 8.60 eV, five different versions of the TPES with different *eKE*_max_, a table with suggestions for vibrational assignments for the electronic spectrum assuming JT splitting comparable to those presented in Tian et al.^69^ for C_70_^3‑^, and a table with tentative assignments and energies for the excited states of the cation which are shown in Figure 6 (PDF)
