# Supplementary material for: Experimental VUV Photoionization of C70 and Vibrationally Resolved Spectra of the Excited Electronic States of the C70 + Cation
Source: ACS Earth Space Chem. 2025 Oct 17;9(11):2694–704. doi: 10.1021/acsearthspacechem.5c00217 (PMC12641481; doi:10.1021/acsearthspacechem.5c00217)
Supplement: Supplementary file 1 [file sp5c00217_si_001.pdf]

# Experimental VUV Photoionization of $C_{70}$ and vibrationally resolved spectra of the excited electronic states of the $C_{70}^+$ cation – Supplementary information

Lisa Ganner[1], Gustavo A. Garcia[2], Martin Schwell[3], Miriam Kappe[1], Laurent Nahon[2], Elisabeth Gruber[1]*, Helgi Rafn Hrodmarsson[3]*

[1] Institute for Ion Physics and Applied Physics, University of Innsbruck, A-6020 Innsbruck, Austria
[2] Synchrotron SOLEIL, L'Orme des Merisiers, 91192 Gif sur Yvette Cedex, France
[3] LISA UMR 7583 Université Paris-Est Créteil and Université de Paris, Institut Pierre et Simon Laplace, 61 Avenue du Général de Gaulle, 94010 Créteil, France

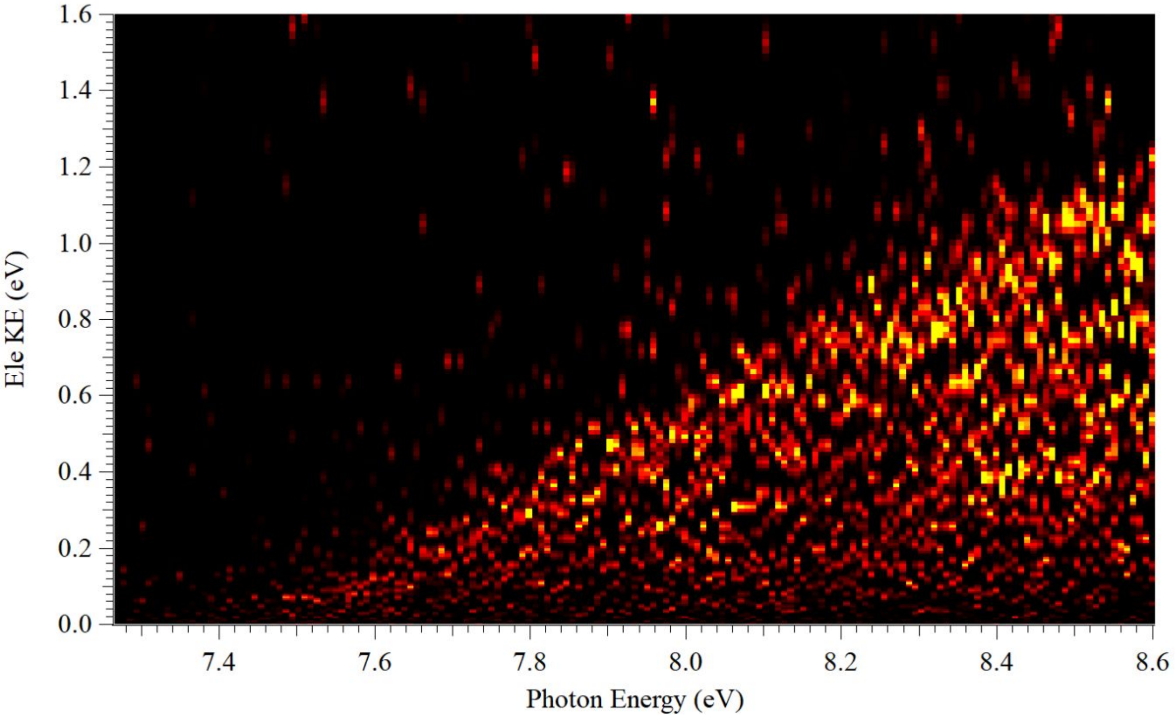


**Figure S1.** Two-dimensional photoelectron spectrum matrix of $C_{70}$.

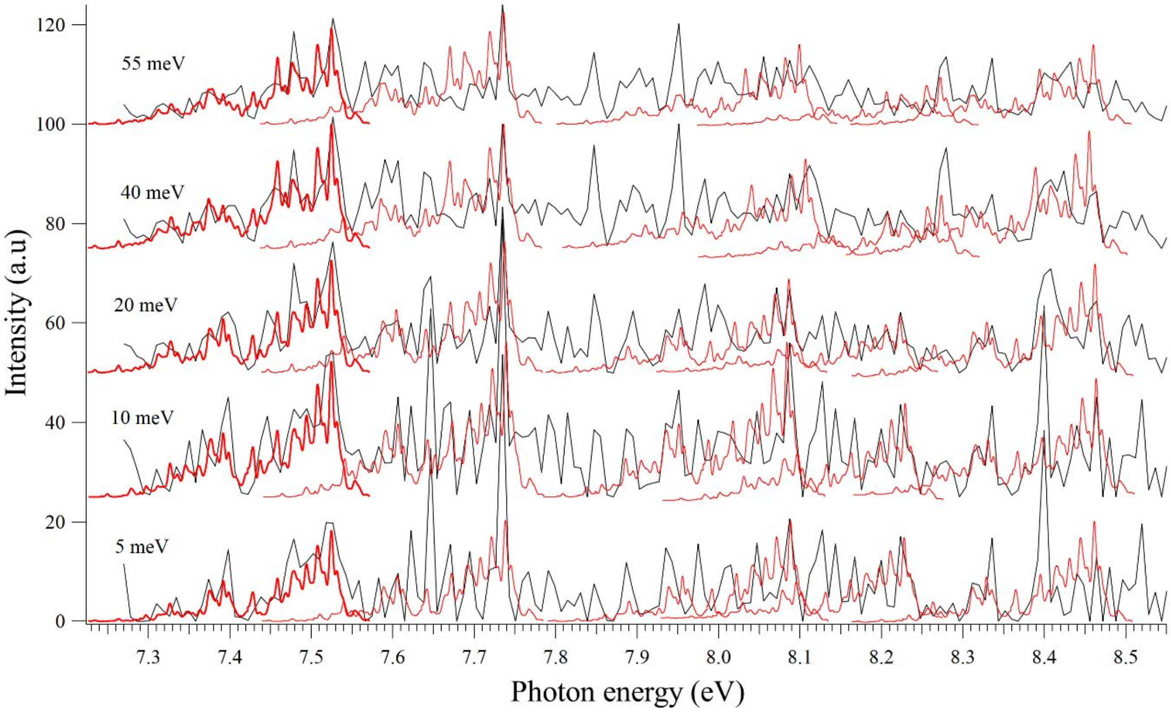


**Figure S2.** TPES with $eKE_{max}$ = 5, 10, 20, 40, and 55 meV. The red bold lines trace the hot band fits created to fit the 0-0 transitions to the ground state. The thin lined red traces are the best matches of the hot band fits to the TPES used to identify the remaining excited states.

**Table S1.** Peak positions and tentative assignments based on the theoretical work of Tian *et al.*[1] of $C_{70}^{3-}$, band energies (Δ), and comparisons to the works of Campbell *et al.*[2] and Tian *et al.*[1] which comprises the three columns on the right. Peaks marked with * should be considered tentative. Combination bands are marked with a plus sign. When two or more fundamental bands could be contributing to a peak we denote them with an ampersand (&).

| Peak number | Cation (This work) | Assignment | Δ | Cation (Campbell) | Δ | $A_1'$ | $E_2'$ | $E_1'$ |
|---|---|---|---|---|---|---|---|---|
| | 12564.0 | 0-0 | 0 | 12564.1 | 0 | 0 | 0 | 0 |
| 1 | 12727.2 | J-T | 163.2 | 12726.5 | 162.4 | | | |
| 2 | 12747.7 | $E_2'\,(v_{21})$ | 183.7 | 12750.1 | 186.0 | | 187 | |
| 3 | 12785.0 | $E_2'\,(v_{20})$ | 220.8 | 12785.6 | 221.5 | | 219 | |
| 4 | 12799.3 | $A_1'\,(v_{12})$ | 235.3 | 12801.0 | 236.9 | 229 | | 240 |
| 5 | 12831.0* | $E_1'\,(v_{21})$ | 267.0 | - | - | | | 275 |
| 6 | 12856.7 | $A_1'\,(v_{11})$ | 292.7 | 12858.1 | 294 | 302 | | |
| 7 | 12874.7 | $E_2'\,(v_{19})$ | 310.7 | 12876.0 | 311.9 | | 317 | 315 |
| 8 | 12904.1 | $E_1'\,(v_{19})$ | 340.1 | 12903.2 | 339.1 | | | 347 |
| 9 | 12939.0 | $A_1'\,(v_{10})$ & $E_2'\,(v_{18})$ | 375 | 12939.8 | 375.7 | 380 | 371 | |
| 10 | 12962.9 | $A_1'\,(v_{12}) + JT$<br>$E_2'\,(v_{21}) + E_2'\,(v_{20})$ | 398.9 | 12964.6 | 400.5 | | | |
| 11 | 12984.6 | $E_2'\,(v_{17})$ & $E_1'\,(v_{18})$<br>$A_1'\,(v_{12}) + E_2'\,(v_{21})$ | 420.6 | 12983.8 | 419.7 | | 413 | 422 |
| 12 | 13012.5 | $A_1'\,(v_9)$ | 448.5 | 13011.0 | 446.9 | 452 | | 439 |
| 13 | 13035.3 | $A_1'\,(v_8)$ & $E_2'\,(v_{16})$<br>$A_1'\,(2v_{12})$ | 471.3 | 13036.6 | 472.5 | 479 | 477 | |
| | | | | | | | 494 | 524 |
| 14 | 13099.7 | $A_1'\,(v_{12}) + A_1'\,(v_{11})$<br>$E_2'\,(v_{14})$&$E_1'\,(v_{16})$&$E_1'\,(v_{15})$ | 535.7 | 13101.7 | 537.6 | | 555 | 538 |
| 15 | 13136.0 | $E_1'\,(v_{14})$ | 572 | - | - | | | 565 |
| 16 | 13152.4 | $A_1'\,(2v_{11})$ | 588.4 | - | - | | | |
| 17 | 13189.3 | $E_2'\,(v_{13})$<br>$E_2'\,(v_{21}) + A_1'\,(v_9)$<br>$E_2'\,(2v_{19})$ | 625.3 | 13188.6 | 624.5 | | 611 | |
| 18 | 13223.8 | $E_2'\,(v_{21}) + A_1'\,(v_8)$<br>$E_2'\,(v_{21}) + E_2'\,(v_{16})$<br>$E_2'\,(v_{19}) + A_1'\,(v_9)$<br>$A_1'\,(v_{12}) + E_2'\,(v_{17})$ | 659.8 | 13230.3 | 666.2 | | | |

| | | | | | | | | | |
|---|---|---|---|---|---|---|---|---|---|
| | | $A_1'\,(v_{12}) + E_1'\,(v_{18})$ | | | | | | | |
| 19 | 13262.8 | $A_1'\,(v_7)\&E_2'\,(v_{12})\&E_1'\,(v_{13})$<br>$E_2'\,(v_{14})\&E_1'\,(v_{16})\&E_1'\,(v_{15}) + JT$<br>$E_2'\,(v_{20}) + A_1'\,(v_8)\ \&\ E_2'\,(v_{16})$ | 698.8 | 13264.9 | 700.8 | 687 | 711 | 686 |
| 20 | 13300.9 | $E_2'\,(v_{11})$<br>$A_1'\,(v_{11}) + A_1'\,(v_9)$ | 736.9 | 13301.4 | 737.3 | | 736 | |
| 21 | 13330.4 | $A_1'\,(v_{11}) + A_1'\,(v_8)$<br>$A_1'\,(v_{11}) + E_2'\,(v_{16})$<br>$E_2'\,(v_{19}) + A_1'\,(v_9)$<br>$E_2'\,(v_{20})$<br>$+ E_2'\,(v_{14})\&E_1'\,($ | 766.4 | 13330.5 | 766.4 | | | |
| 22 | 13370.8 | $E_2'\,(v_{21}) + E_2'\,(v_{13})$<br>$E_2'\,(v_{21}) + E_2'\,(2v_{19})$<br>$E_2'\,(v_{20}) + A_1'\,(2v_{11})$ | 806.8 | | | | | |
| 23 | 13386.2 | $A_1'\,(v_{12}) +\ A_1'\,(2v_{11})$ | 822.2 | 13386.5 | 822.4 | | | |
| 24 | 13406.2 | $E_2'\,(v_{10})$<br>$E_2'\,(2v_{17})\ \&\ E_1'\,(2v_{18})$ | 842.2 | - | - | | 846 | |
| 25 | 13427.4 | $A_1'\,(v_7)\&E_2'\,(v_{12})\&E_1'\,(v_{13}) + JT$<br>$A_1'\,(v_{12}) + E_2'\,(v_{13})$<br>$A_1'\,(v_{12}) + E_2'\,(2v_{19})$<br>$E_1'\,(v_{13})$<br>$E_2'\,(v_{17}) + A_1'\,(v_9)$ | 863.6 | - | - | | | 875 |
| 26 | 13454.6 | $A_1'\,(2v_9)$<br>$E_2'\,(v_{17}) + A_1'\,(2v_{12})$ | 890.6 | - | - | | | |
| | | | | | | | 939 | 933 |
| 27 | 13562.7 | $A_1'\,(v_{10}) + E_2'\,(v_{13})$<br>$E_2'\,(v_{18}) + E_2'\,(v_{13})$<br>$A_1'\,(v_{10}) + E_2'\,(2v_{19})$<br>$E_2'\,(v_{18}) + E_2'\,(2v_{19})$ | 998.7 | 13563.4 | 999.3 | | | |

**Table S2.** Assignments of 0-0 transitions to the ground states and excited states and comparison to the work of Lichtenberger *et al.*[3]

| 0-0 Assignment | This work | Lichtenberger *et al.* (1992) |
|---|---|---|
| $^2E_1''$ | 7.429 ± 0.015 | 7.47 |
| $^2A_2'$ | 7.639 ± 0.015 | 7.68 |
| $^2E_2'$ | 7.992 ± 0.015 | 7.96 |
| $^2E_2''$ | 8.184 ± 0.015 | 8.12 |
| $^2E_1'$ | 8.368 ± 0.015 | 8.43 |

## References


(1) Tian, L.; Yi, Y.-S.; Wang, C.-L.; Su, Z.-B. E⊗e Jahn–Teller Effect in $C_{70}^{3-}$ Systems. *Int. J. Mod. Phys. B* **1997**, *11* (16), 1969–1978. https://doi.org/10.1142/S0217979297001039.

(2) Campbell, E. K.; Holz, M.; Maier, J. P.; Gerlich, D.; Walker, G. A. H.; Bohlender, D. Gas Phase Absorption Spectroscopy of $C_{60}^{+}$ and $C_{70}^{+}$ in a Cryogenic Ion Trap: Comparison with Astronomical Measurements. *Astrophysical Journal* **2016**, *822* (1). https://doi.org/10.3847/0004-637X/822/1/17.

(3) Lichtenberger, D.; Rempe, M.; Gogosha, S. The He-I Valence Photoelectron-Spectrum of $C_{70}$ in the Gas-Phase. *Chem. Phys. Lett.* **1992**, *198* (5), 454–460. https://doi.org/10.1016/0009-2614(92)80027-9.
